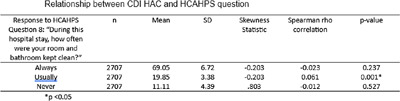# Does Patient Perception of Cleanliness Still Matter? The Relationship between HCAHPS and HAC During the COVID-19 Pandemic

**DOI:** 10.1017/ash.2024.301

**Published:** 2024-09-16

**Authors:** Caitlin Crews-Stowe

**Affiliations:** University of Tennessee at Chattanooga

## Abstract

**Background:** Environmental contamination of surfaces is known to be one of the most common causes of healthcare associated infections (HAIs), with cleaning and disinfection of surfaces shown to reduce the incidence of HAIs and contribute to overall hospital cleanliness. Prior research showed that there was a relationship between a hospital’s performance on the Hospital Consumer Assessment of Healthcare Providers and Systems survey question on patient perception of cleanliness and their HAC score. However, this research was done prior to the COVID-19 pandemic. This study looked to examine if the pandemic changed the relationship between patient perception of cleanliness and HAC score performance. **Method:** A retrospective correlational study was performed to examine if the relationship between patient perception of cleanliness and the incidence of Clostridioides difficile (CDI) and Methicillin-Resistant Staphylococcus aureus (MRSA) HAIs, as defined by the facility’s HAC score, were affected by the COVID-19 pandemic. Multiple Center for Medicare and Medicaid Services (CMS) datasets were utilized for the study. There were approximately 2700 acute care facilities that reported data on the HCAHPS perception of cleanliness question and either a MRSA or CDI HAC score for the period of January 1, 2021, to December 31st, 2021. Basic descriptive statistics and Spearman’s rho correlation analyses were performed to examine the potential associations between the two scores. **Result:** For MRSA, the study found that as the percentage of patients who reported that their room was “always” clean increased, the hospital’s HAC score decreased (r= −0.141, p = < 0.001). Additionally, as the percentage of patients who reported their room was “never” clean increased, the hospital’s HAC score increased (r= 0.175, p = <0.001) For C. difficile, the analysis also revealed that as the percentage of patients who reported their room was “always” clean increased, there was not a significant change in the hospital’s HAC score (r= −0.023, p = 0.237). There was also not a significant change in the HAC score when the percentage of patients who reported their room as “never” clean increased (r= −0.012, p = 0.527).

**Conclusion:** The study found that for MRSA, a hospital’s performance on the HCAHPS performance on patient perception of cleanliness is related to their performance on their HAC score. This did not hold true when looking at C. difficile infections, which is in contrast to the prior evidence. Further research is needed to determine if there are specific factors that may have influenced this change.

**Disclosure:** Caitlin Crews-Stowe: Employee- ActivePure Medical

**Figure:**
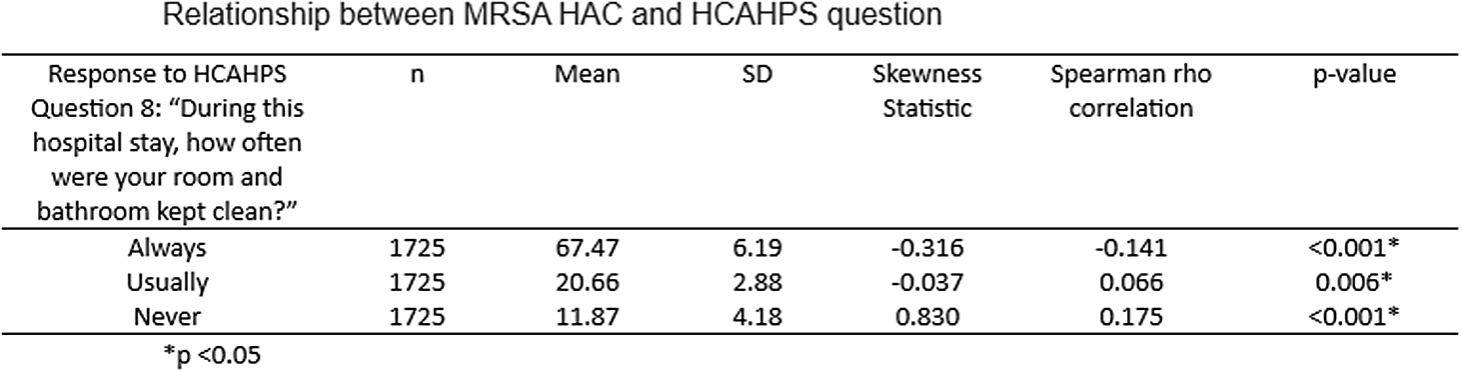

**Figure:**